# Influence of Alkali Metal Cations on the Oxygen Reduction Activity of Pt_5_Y and Pt_5_Gd Alloys

**DOI:** 10.1021/acs.jpcc.4c00531

**Published:** 2024-03-18

**Authors:** Kun-Ting Song, Alexandra Zagalskaya, Christian M. Schott, Peter M. Schneider, Batyr Garlyyev, Vitaly Alexandrov, Aliaksandr S. Bandarenka

**Affiliations:** † Physik-Department ECS, Technische Universität München, James-Franck-Str. 1, Garching D-85748, Germany; ‡ Department of Chemical and Biomolecular Engineering, University of Nebraska-Lincoln, Lincoln, Nebraska 68588, United States; § Quantum Simulations Group, Materials Science Division, Lawrence Livermore National Laboratory, Livermore, California 94550, United States; ∥ Nebraska Center for Materials and Nanoscience, University of Nebraska-Lincoln, Lincoln, Nebraska 68588, United States; ⊥ Catalysis Research Center TUM, Ernst-Otto-Fischer-Straße 1, Garching bei München 85748, Germany

## Abstract

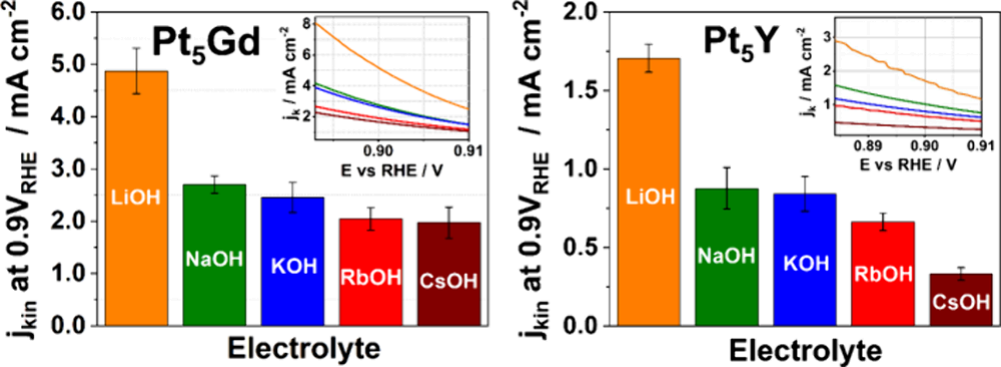

Electrolyte species can significantly influence the electrocatalytic performance. In this work, we investigate the impact of alkali metal cations on the oxygen reduction reaction (ORR) on active Pt_5_Gd and Pt_5_Y polycrystalline electrodes. Due to the strain effects, Pt alloys exhibit a higher kinetic current density of ORR than pure Pt electrodes in acidic media. In alkaline solutions, the kinetic current density of ORR for Pt alloys decreases linearly with the decreasing hydration energy in the order of Li^+^ > Na^+^ > K^+^ > Rb^+^ > Cs^+^, whereas Pt shows the opposite trend. To gain further insights into these experimental results, we conduct complementary density functional theory calculations considering the effects of both electrode surface strain and electrolyte chemistry. The computational results reveal that the different trends in the ORR activity in alkaline media can be explained by the change in the adsorption energy of reaction intermediates with applied surface strain in the presence of alkali metal cations. Our findings provide important insights into the effects of the electrolyte and the strain conditions on the electrocatalytic performance and thus offer valuable guidelines for optimizing Pt-based electrocatalysts.

## Introduction

The role of renewable energy in developing sustainable economies is increasingly important.^1,2^ Energy storage and conversion devices such as metal-air batteries and polymer electrolyte membrane fuel cells (PEMFCs) are necessary to achieve this goal.^3−8^ However, several critical problems must be solved to efficiently employ these devices. For example, in the case of the oxygen reduction reaction (ORR) in fuel cells, sluggish reaction kinetics increase the cathode mass loading and limit the overall performance of PEMFCs.^9,10^ Thus, finding more efficient electrocatalysts to enhance the ORR performance is critical. Platinum (Pt) is commonly used as an ORR catalyst. Still, it does not reach optimal catalytic activity on the Sabatier volcano plot due to the non-optimum binding of key reaction intermediates such as *OOH, *O, and *OH at the electrode/electrolyte interface according to density functional theory (DFT) calculations.^11−14^ Additionally, the scarcity and high cost of raw materials make commercial use of Pt as a catalyst problematic.^15−18^ Therefore, strategies to decrease the mass loading and enhance the catalytic performance of Pt-based catalysts are crucial for commercializing PEMFCs.^19−21^

Efforts to develop higher-performance PEMFCs have been largely focused on the optimization and tailoring of the shape, size, and surface structure of Pt-based electrocatalysts.^22−25^ Electrochemical scanning tunneling microscopy (EC-STM) has often been used to identify the nature of active sites that provide optimal binding energy for reaction intermediates during ORR.^26−28^ This allows one to explore the correlation between the coordination and the adsorption energies of intermediate species on active sites and maximize their number in nanostructured electrocatalysts.^25^ Furthermore, alloying Pt with lanthanide materials and transition metals is a common approach to modify surface chemical properties generally referred to as strain and/or ligand effects.^29−31^ Such an alloying may also improve ORR activity in polycrystalline and nanoparticulate catalysts.^10,28,29,32−38^ The Pt-enriched overlayer forms on the bulk of Pt alloys after the so-called acid-leaching process.^10^ The consequent lattice mismatch between the acid-leached Pt overlayer and the bimetallic bulk alloy causes in the overlayer. The ligand effects are attributed to the dissimilar surrounding atoms, which influence the electronic structure as well as the surface chemical properties.^30^ The combination of strain and ligand effects results in weaking of the binding energy of intermediates and improves the ORR activity.

Furthermore, electrolyte effects in electrocatalytic systems have become an active area of research in recent years. The anion and cation species from supporting electrolytes are regarded as “spectators”, which can effectively affect the electrode/electrolyte interfacial properties and electrocatalytic performance.^39−44^ Frumkin initially demonstrated the correlation between cation adsorption and reaction kinetics within the double-layer region.^45^ Strmcnik et al. illustrated that the noncovalent interactions between reaction intermediates and hydrated alkali metal cations alter the reaction kinetics at the electrode/electrolyte interface.^43^ In the case of Pt(111) electrodes, the ORR activity in alkaline media has been examined,^43^ and the catalytic trend is CsOH > KOH> NaOH > LiOH. Moreover, Huang et al. reported an opposite trend for Pt polycrystalline (Pt(pc)) toward hydrogen evolution and oxidation reactions (HER/HOR) in alkaline media,^46^ which further highlighted the fact that cation-dependent interfacial hydrogen-bonding network can strongly affect reorganization energy and reaction entropy.

Herein, the main objective of this work is to investigate the role of alkali metal cations in the ORR activity of Pt-based alloys in the presence of induced strain on the surface. Here, we select Pt_5_Gd and Pt_5_Y polycrystalline alloys as the model electrodes for Pt-based alloys due to their high thermodynamic stability with negative heat formation to prevent the continuous dealloying process and provide a relatively steady system during reactions.^32,35,47,48^ We measured the strain effects on the ORR activity of Pt alloys and further investigated the cation effect on the activities under strain conditions in different alkaline media. Besides the experimental findings, we employed DFT calculations for the corresponding model systems to study the correlation between the ORR theoretical overpotential and the adsorption energies of the intermediates accounting for both strain and cation effects.

## Methods

### Experimental Section

The electrodes of Pt(pc), Pt_5_Gd, and Pt_5_Y (MaTeck, Germany) with a diameter of about 5 mm were utilized as working electrodes (WEs) in this work. For the electrochemical measurements, the diluted perchloric acid solution (70% HClO_4_, Suprapur, Merck, and extra pure, Acros, Germany) is selected for the acidic media. The diluted alkaline solutions are synthesized by lithium hydroxide (LiOH, anhydrous, 99.995% (metals basis), Thermo Fisher Scientific, USA), sodium hydroxide (NaOH, 99.99% (metals basis), Thermo Fisher Scientific, USA), potassium hydroxide (KOH, 99.98% (metals basis), Alfa Aesar, USA), rubidium hydroxide (RbOH solution, 50 wt % in H_2_O, 99.9% trace metals basis, Sigma-Aldrich, USA), and cesium hydroxide (CsOH solution, 50 wt % in H_2_O, 99.9% trace metals basis, Sigma-Aldrich, USA). Ultrapure water (18.2 MΩ·cm, Merck Millipore, Germany) was used to prepare all diluted solutions.

Besides, X-ray diffraction (XRD) was used to investigate the structural and crystallographic properties of electrodes. The XRD measurements were conducted by X’Pert pro PANanalytical, including a Cu-Ka source and a Ni-based filter. The scanning range (2θ) was from 5° to 90° with a scan rate of approximately 0.78° min^–1^. The crystal structure for the electrodes, Pt(pc), Pt_5_Gd, and Pt_5_Y, were fitted with the powder diffraction files corresponding to the literature.^10,32,35^ X-ray photoelectron spectroscopy (XPS) was conducted to analyze the surface composition of the Pt alloys after electrochemical measurements by Thermo Scientific K-Alpha^+^. The XPS spectra were fitted by the CasaXPS software.

Prior to the electrochemical measurements, the cells were cleaned with the so-called “Piranha solution”, a 3:1 mixture of sulfuric acid (96% H_2_SO_4_, p.a., ISO, Carl Roth, Germany) and hydrogen peroxide (30% H_2_O_2_, p.a., ISO, Carl Roth, Germany), and rinsed with hot ultrapure water several times. The electrochemical cell was composed of a WE, a Pt wire (99.9% purity, MaTeck, Germany) as a counter electrode (CE), and a mercury-mercurous sulfate (MMS) (SI Analytics, Germany) electrode or a mercury-mercuric oxide (MMO) (BAS Inc., Japan) as a reference electrode (RE). For the activity measurements of the ORR in acidic and alkaline media, the cyclic voltammograms (CVs) were first recorded in Ar-saturated conditions as the background CVs for the ORR polarization curves with a scan rate of 50 mV s^–1^ for the Pt_5_Gd, Pt_5_Y, and Pt(pc) electrodes. After reaching stable CVs, the ORR measurements were then collected in the O_2_-saturated condition at the rotation speed rate of 1600 rpm with a scan rate of 50 mV s^–1^. In this work, all voltammograms of ORR are presented with the *iR*-correction potential due to the ohmic drop losses, and the current is subtracted from the background current in Ar-saturated CVs. The ORR kinetic current density is determined from the following Koutecký–Levich equation.^49^
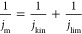
where *j*_m_, *j*_kin_, and *j*_lim_ represent the measured, the kinetic, and the diffusion-limited current density, respectively.

The ohmic drop, mainly due to the solution and electric resistance of the three-electrode setup, was measured via electrochemical impedance spectroscopy (EIS) with a shunt capacitor connected between RE and CE to filter out the error signal from the high-frequency range.^50^ All electrochemical measurements were conducted via a VSP-300 potentiostat (Bio-Logic, France).

It is noted that all measured potentials with the REs of MMS or MMO were converted to the reversible hydrogen electrode (RHE) scale by the potential calibration in the H_2_-saturated condition to avoid the potential shift due to the liquid junction potential appearing between the inner electrolyte and the operating electrolyte.^51,52^ The solutions were purged with H_2_ for about 30 min, and the CVs were recorded with a scan rate of 10 mV s^–1^ at 1600 rpm to find the intersection potentials of zero current and to obtain the average potential for the measured acidic and alkaline solutions. It is worth emphasizing that the calibration process is necessary to estimate the ORR performance in different solutions precisely. Furthermore, to make sure that the DURAN borosilicate glass cell used for the ORR measurements in alkaline solutions has no impact on the activities due to undesired contamination issues from the glass in alkaline media,^53,54^ all measurements were conducted in short-term periods in diluted alkaline solutions. The self-constructed perfluoroalkoxy (PFA) electrochemical cell was used to compare with a typical glass cell.

Finally, to precisely estimate the electrochemical active surface area (ECSA) for the Pt_5_Gd, Pt_5_Y, and Pt(pc) electrodes, the Cu underpotential deposition measurements were carried out according to the method illustrated by Green and Kucernak^55^ and Stephens et al.,^35^ which are suitable for determining the ECSA of Pt-based alloys. Before the Cu underpotential deposition measurements, the electrode surface was first cleaned electrochemically in Ar-saturated 0.1 M HClO_4_ until stable CVs were reached. The electrode was then measured in an Ar-saturated mixture solution of 0.1 M HClO_4_ and 2 mM CuO in the hanging meniscus configuration. The WEs were polarized at 1.0 V vs RHE for 160 s to remove Cu before being polarized at 0.3 V vs RHE for 100 s to form a Cu monolayer on the surface. The CVs were recorded in the potential range from 0.06 to 1.0 V vs RHE and from 0.3 to 1.0 V vs RHE with the scan rate of 20 mV s^–1^, before and during the Cu underpotential deposition process, respectively. All of the detailed results are shown in the Supporting Information.

### Computational Section

DFT calculations were performed using the revised Perdew–Burke–Ernzerhof (RPBE) functional^56,57^ in Vienna Ab Initio Simulation Package (VASP).^58,59^ The projector-augmented wave (PAW) potentials were taken from the VASP library (Pt, O, H, Li_sv, Na_sv, K_sv, Rb_sv, and Cs_sv). The unit cell of bulk Pt was optimized with a 9 × 9 × 9 Monkhorst–Pack mesh to sample the *k*-space and cutoff energy of 700 eV. The convergence criteria for the total energy and atomic forces were set to 10^–8^ eV and 0.01 eV/Å^–1^, respectively. The computed Pt lattice parameter of 3.9974 Å differs from the experimental value of 3.9237 Å^35^ by 2%, which is related to overbinding in RPBE but allows one to obtain more accurate binding energies.^32,60^ In this paper, we refer our results to the computed values, and therefore, experimental data correspond to the strain of −2%.

The six-layer Pt(111) slab was constructed in VESTA^61^ with a vacuum gap of at least 20 Å applying tensile and compressive strain. When applying tensile and compressive strains, the coordinates of all Pt atoms were fixed to avoid surface reconstruction leading to the local strain that would not correspond to the targeted strain value.

All alkali cations were placed on the most favorable fcc site, while the top position was considered for all of the ORR intermediates. The computational hydrogen electrode (CHE) approach^11,62^ was employed to calculate the free energies of *O, *OH, and *OOH intermediates, including zero-point energy (ZPE) and vibrational entropy *S*_vib_ contributions taken at *T* = 300 K as ΔG_*X_ = E_*X, DFT_ + ZPE – *TS*_vib_, where *X stands for the above-mentioned reaction intermediates. The following corrections of 0.36, 0.07, and 0.40 eV were taken to include the zero-point energy and vibrational entropy contributions for *OH, *O, and *OOH. Adsorption energies of the reaction intermediates were computed as ΔG_*OH, ads_ = ΔG_*OH_ – ΔG_*_ – Δ*G*_H2O_ – 1/2Δ*G*_H2_; ΔG_*O, ads_ = ΔG_*O_ – ΔG_*_ – Δ*G*_H2O_ – Δ*G*_H2_; ΔG_*OOH, ads_ = ΔG_*OH_ – ΔG_*_ – 3/2Δ*G*_H2O_ – 2Δ*G*_H2_.

The theoretical overpotential of the ORR was computed as η = 1.23 + max {Δ*G*_1_, Δ*G*_2_, Δ*G*_3_, and Δ*G*_4_}/*e*, where Δ*G*_1_ = ΔG_*OOH_ – 4.92, Δ*G*_2_ = ΔG_*O_ – ΔG_*OOH_, Δ*G*_3_ = ΔG_*OH_ – ΔG_*O_, and Δ*G*_4_ = −ΔG_*OH_.

A plane-wave cutoff energy of 450 eV and a 3 × 3 × 1 Monkhorst–Pack sampling of the reciprocal space were adopted in all slab calculations. The convergence criteria for the total energy and atomic forces were set to 10^–4^ eV and 0.05 eV/Å, respectively.

## Results and Discussion

The Pt-based electrocatalysts, including Pt_5_Gd, Pt_5_Y, and Pt(pc) electrodes, were first characterized by XRD measurements. As shown in Figure S1 and Table S1, all XRD patterns show typical polycrystalline structures of all measured electrodes, and the patterns are comparable to the literature with the corresponding crystalline structures and lattice parameters.^10,32,35^ Moreover, the XPS results in Figure S2 prove the formation of a Pt overlayer on the surface of bulk Pt_5_Gd and Pt_5_Y electrodes after the electrochemical measurements. The surface compressive strain is introduced on the Pt overlayer of bulk Pt alloys after the acid leaching process due to the different lattice constants between the surface and bulk crystal.

**Figure 1:**
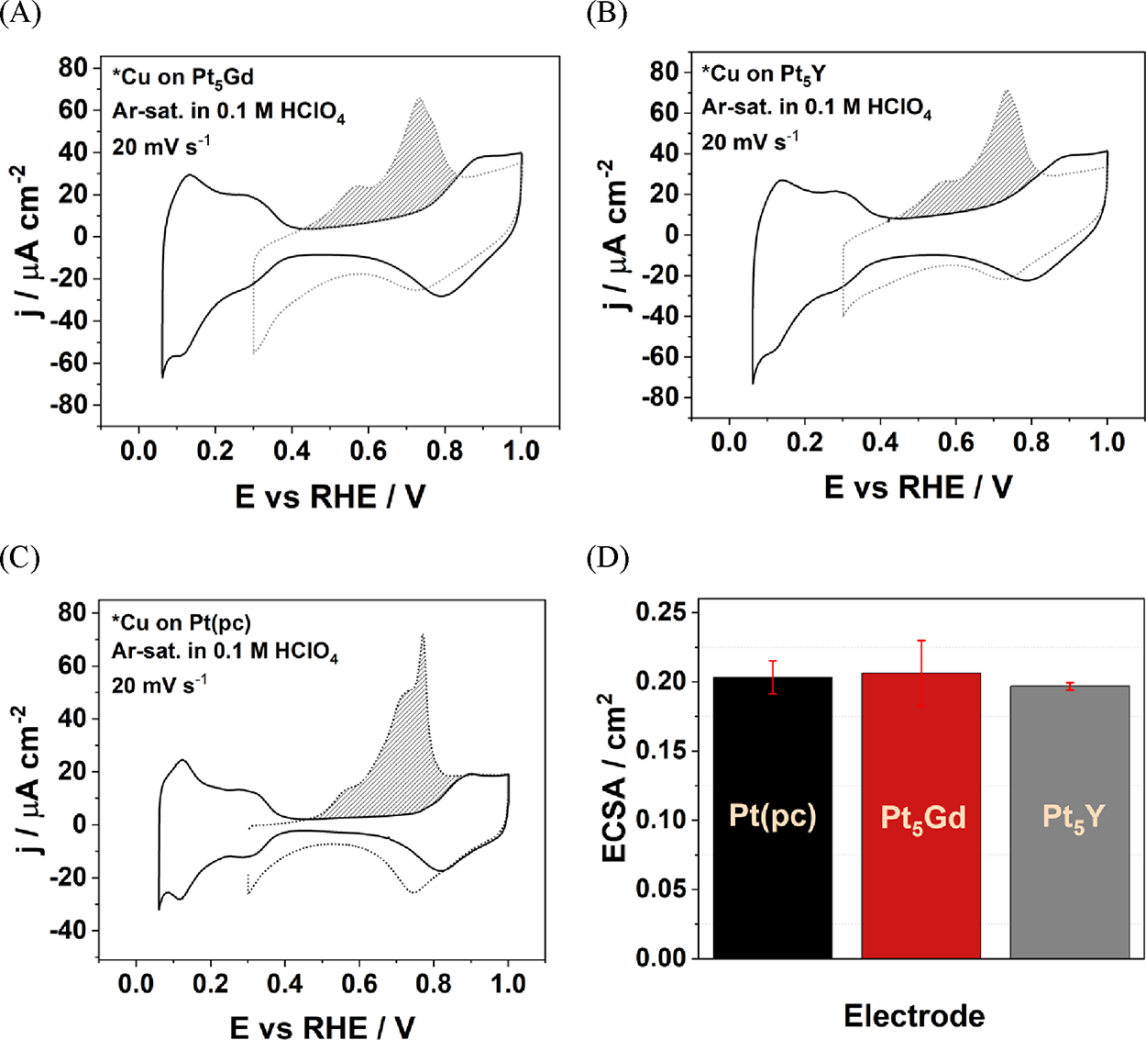
Typical CVs of (A) Pt_5_Gd, (B) Pt_5_Y, and (C) Pt(pc) electrodes in Ar-saturated 0.1 M HClO_4_ with the scan rate of 20 mV s^–1^. The solid and dashed lines represent the typical CVs and the first cycle of Cu stripping, respectively, with the estimated electrochemical active surface areas noted in gray. (D) The bar chart represents the ECSAs of Pt_5_Gd, Pt_5_Y, and Pt(pc) electrodes determined by Cu underpotential deposition/monolayer stripping.

To study electrochemical properties, CVs of bulk Pt_5_Gd, Pt_5_Y, and Pt(pc) electrodes were recorded in Ar-saturated 0.1 M HClO_4_ within the potential range between 0.06 and 1.0 V vs RHE in Figure 1A–C, respectively. Furthermore, the ECSA was estimated by the Cu underpotential deposition and subsequent monolayer stripping method. It is noted that this approach has some advantages for bimetallic electrocatalysts because it can precisely distinguish the contribution sites of deposited Cu atoms from Pt sites with no possible contribution of the solute atoms with different adsorption energies at the surface.^28,55^Figure 1D shows that the average ECSA of each bulk electrode is approximately 0.2 cm^2^, similar to their geometric surface area (for an approximately 5 mm diameter disk). Evaluation of the anodic peaks between the Cu underpotential deposition and the background CVs (area with gray color) yields an integrated charge density of about 420 μC cm^–2^, which is close to the ideal assumption of a monolayer of *Cu on a pure Pt surface and further verifies the formation of a Pt overlayer on bulk Pt alloys (where “*” is noted as the adsorption site of an atom). As the ECSA of each measured electrode is comparable to the geometric surface area, all recorded currents in this work are normalized to the geometric surface area.

**Figure 2:**
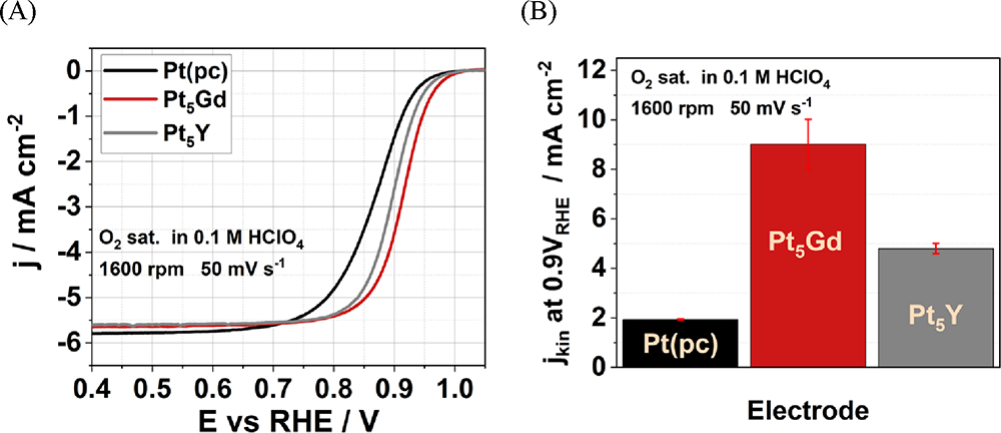
(A) *iR*-corrected voltammograms with anodic scan and (B) bar chart of the *j*_kin_ at 0.9 V vs RHE of the Pt(pc), Pt_5_Gd, and Pt_5_Y electrodes in O_2_-saturated 0.1 M HClO_4_ with the scan rate of 50 mV s^–1^ at 1600 rpm.

To assess the ORR performance in an acidic environment, the standard ORR polarization curves in an O_2_-saturated 0.1 M HClO_4_ solution at 1600 rpm were recorded (see Figure 2A). The trend of the extracted ORR kinetic current densities at 0.9 V vs RHE shown in Figure 2B is as follows: Pt_5_Gd > Pt_5_Y > Pt(pc). The measured ORR activities for each system are found to be comparable to the literature data.^10,32,35,36^ The 2–4 times enhanced ORR activities of Pt alloys compared to pure Pt electrodes are attributed to the effect of compressive strain in the Pt overlayer on the surface, where the surface Pt–Pt interatomic distances decrease compared to the bulk Pt alloys as seen from XRD measurements in Figure S1 and Table S1 and from the literature.^10,32,35^ The results of increased ORR activities for Pt alloys are also supported by the d-band theory.^48,63^ For Pt-overlayers under compressive strains, the metal d-band center energies shift downward. The binding energy of adsorbed intermediates, including *O, *OH, and *OOH, on the Pt overlayer becomes lower due to the compressive strains. The ORR performance improves to reach the optimal state, where the binding energy is approximately 0.2 eV lower than on pure Pt.^63,64^

**Figure 3:**
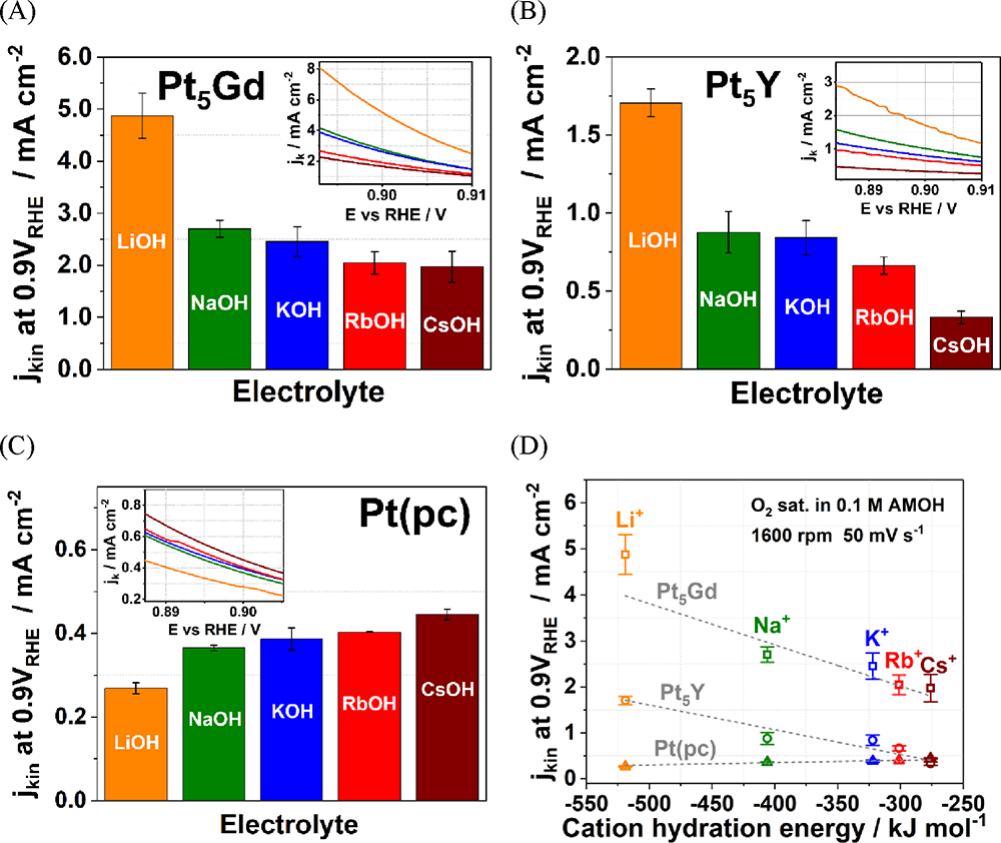
*iR*-corrected voltammograms with anodic scan of (A) Pt_5_Gd, (B) Pt_5_Y, and (C) Pt(pc) electrodes in O_2_-saturated 0.1 M AM–OH (AM^+^ = Li^+^, Na^+^, K^+^, Rb^+^, and Cs^+^) electrolytes with the scan rate of 50 mV s^–1^ at 1600 rpm. The insets in panels (A–C) represent exemplary *j*_kin_ versus potential curves. (D) The *j*_kin_ at 0.9 V vs RHE of the Pt(pc), Pt_5_Gd, and Pt_5_Y electrodes was determined from the insets as a function of cation hydration energy with the dashed lines of the linear fit.

To understand the influence of alkali metal cations on the ORR performance, the bar charts and the inset polarization curves shown in Figure 3A–C illustrate the ORR kinetic current densities at 0.9 V vs RHE measured in the O_2_-saturated 0.1 M metal hydroxide electrolytes, AM–OH (AM^+^ = Li^+^, Na^+^, K^+^, Rb^+^, and Cs^+^) for all electrodes under the same experimental conditions as in acid shown in Figure 2A. The ORR activities measured in alkaline media are several factors lower than in acid for all electrodes, and the results are comparable to the previous studies of Pt-based catalysts.^65−67^ One of the reasons for lower ORR activities in alkaline media may be due to the unwarranted hydroperoxyl (HOO^–^) species formed on the electrode surface with the undesired 2e^–^ reaction pathway.^68,69^ Another explanation is that the different reaction mechanisms of ORR in an alkaline solution with too strong Pt–OH bonding energy are possible, which undesirably blocks the Pt active surface.^68^

Moreover, we determine for the Pt_5_Gd and Pt_5_Y systems in alkaline solutions (see Figure 3A,B) that the largest ORR kinetic current densities correspond to LiOH, which is more than twice greater than those measured in CsOH. However, the trend is reversed for Pt(pc), for which the highest ORR activity is observed in the case of CsOH, as seen in Figure 3C. These observations can be correlated with the noncovalent interactions between the hydrated alkali metal cations in the outer Helmholtz plane (OHP), as well as the adsorbed and reactive oxygen species in the inner Helmholtz plane (IHP) at the solid/liquid interface as reported in ref (43). The greater structure-making cations (Li^+^ and Na^+^) strongly bind and interact with the chemisorbed species on pure Pt surfaces with no strain. As a result, besides the intrinsic stronger binding energy like Pt–OH, the extrinsic alkali cations with higher solvation energy inhibit the movement of reactive species and impede the ORR reaction kinetics. By contrast, the compressed-strain surface may have weaker binding energy with the adsorbed species for the Pt overlayer on bulk alloys. Stronger solvation shells optimize and stabilize the Helmholtz plane’s molecular interaction energy and promote the ORR process than weaker solvated cations (Rb^+^ and Cs^+^). Figure 3D shows the ORR kinetic densities at 0.9 V versus RHE as a function of cation hydration energy for the studied electrodes. The ORR activities of Pt alloys decline linearly with the decrease of the hydration energy for alkali cations in the order of Li^+^ > Na^+^> K^+^ > Rb^+^ > Cs^+^. Conversely, Pt(pc) electrodes display the opposite trend.

**Figure 4:**
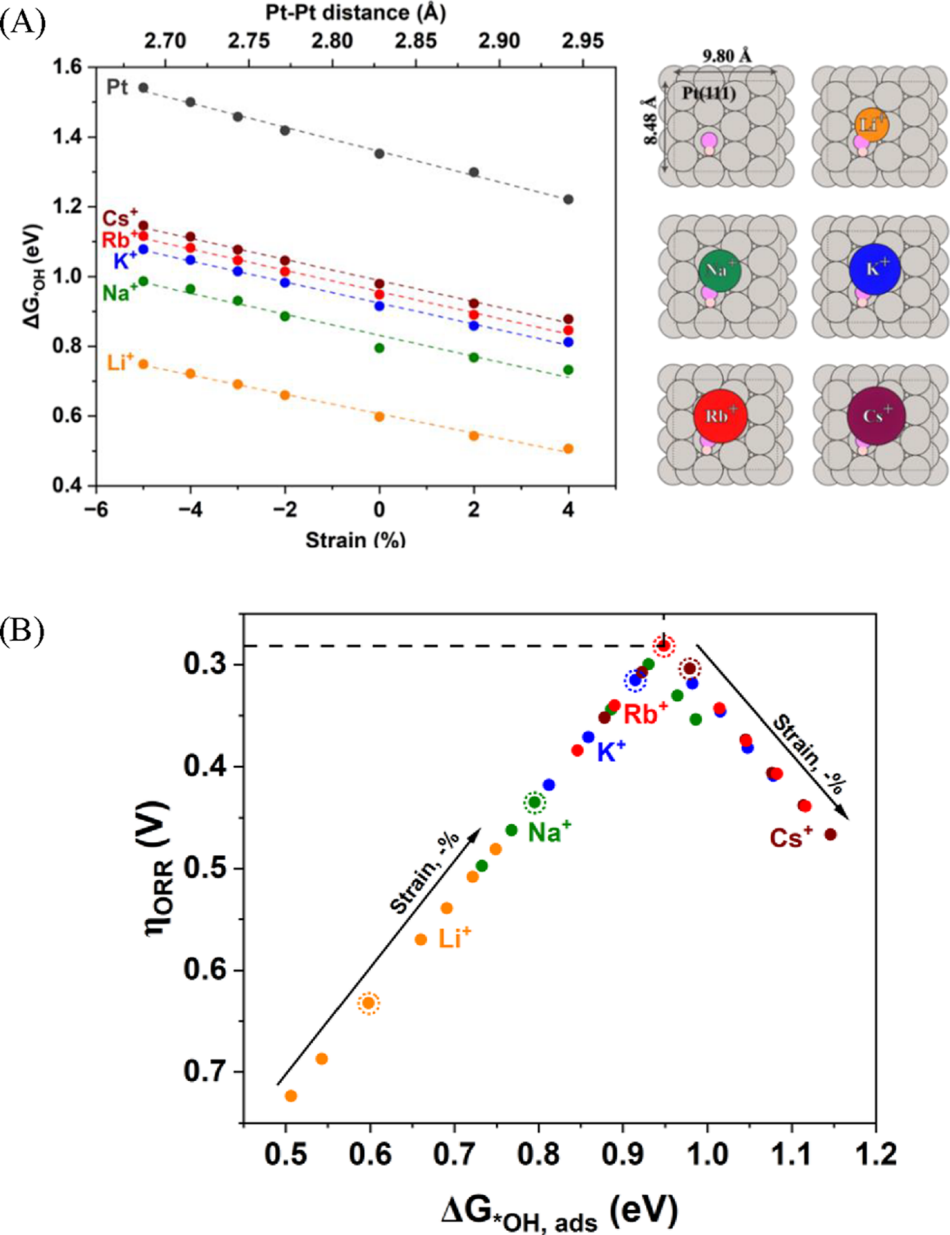
(A) The adsorption energy of *OH on Pt(111) as a function of strain ranged from −5% (compression) to +4% (expansion). The schematic structures and cell dimensions corresponding to 0% strain are shown on the right. (B) The theoretical volcano plot represents the relationship between ORR activity and computed *OH adsorption energy on the pristine Pt(111) surface and in the presence of alkali metal cations. The arrows show the direction of the strain (from tensile to compressive), and the encircled dots correspond to 0% strain. Dashed lines depict the minimum calculated overpotential of 0.28 V corresponding to Rb^+^-Pt@0%. Details of the performed calculations can be found in the Supporting Information.

The observed opposite ORR trends for the Pt catalysts in alkaline electrolytes are further investigated using DFT calculations. Here, we consider the Pt(111) surface slab with an adsorbed *OH species as the model ORR electrocatalytic system. The Pt(111) surface is commonly taken as a model for Pt electrodes due to its low surface energy and the common existence within the crystalline structures of metals.^32,70^ The applied strain corresponding to the surface Pt–Pt distance is illustrated in Table S3. Figure 4A shows the difference between the *OH adsorption energy for the system under applied strain and the value obtained for the case with 0% strain. It is seen that compressive strain leads to the destabilization of *OH on the pristine Pt surface,^32,71^ as well as in the presence of alkali metal cations. This was previously demonstrated to improve the ORR activity as the destabilization of *OH on pure Pt(111) moves ΔG_*OH_ closer to the volcano peak. The obtained slope of −0.03 eV/% for pure Pt fully agrees with previous computational results.^32^ Contrary to the destabilizing effect of the compressive strain on *OH binding, alkali cations stabilize *OH adsorption. However, different alkali cations stabilize *OH on the surface differently, with Li^+^ having the strongest stabilizing effect. For example, Li^+^ stabilizes *OH by 0.75 eV, while Cs^+^ stabilizes *OH by 0.37 eV relative to the pristine Pt surface (see Tables S4 and S5).

As seen in Figure 4B, to achieve the minimum of the ORR theoretical overpotential (η_ORR_), the effects of both strain and the nature of the alkali cation should be considered. Specifically, along with the smaller compressive and nonstrain region (i.e., pure Pt surface, where *OH adsorption is too strong), Cs^+^ is characterized by lower computed ORR overpotentials than Li^+^ as Li leads to larger stabilization of *OH. For example, for the pristine Pt@0%, η_ORR_ (Li^+^) = 0.63 V > η_ORR_ (Cs^+^) = 0.30 V. When applying larger compressive strains (i.e., corresponding to the Pt overlayer on bulk alloys), the ORR overpotential in the presence of Li^+^ decreases. In contrast, the opposite trend is observed for Cs^+^. This is because under such large compressive strains *OH becomes too destabilized, and the strong stabilizing effect of Li^+^ will bring it closer to the volcano peak with optimal ORR activity. Thus, our computational results support experimental findings on the reverse trend of alkali cations, shown in Figure 3D.

**Figure 5:**
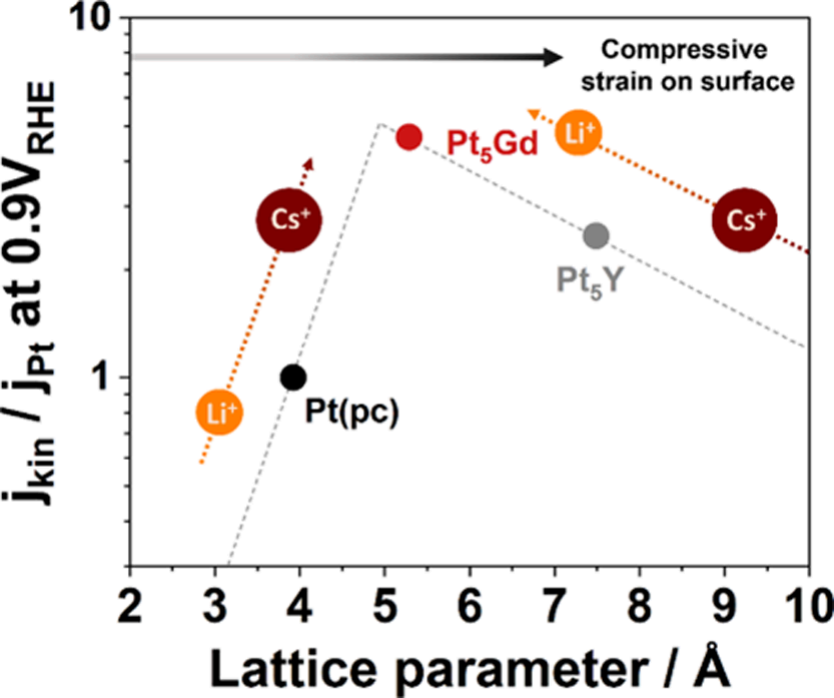
Relative ORR activity at 0.9 V vs RHE as a function of lattice parameter in O_2_-saturated 0.1 M HClO_4_ for Pt(pc), Pt_5_Gd, and Pt_5_Y electrodes (corresponding to black, red, and gray circles, respectively) and their ORR activity-trend measured in 0.1 M O_2_-saturated alkaline solutions with dashed arrows. The induced strain (compression) on the surface increases with a larger lattice parameter. It is noted that the dashed lines of the pseudo volcano plot only represent the guides to the eyes, and the lattice parameters of measured electrodes are referred to the experimental XRD patterns in the Supporting Information in agreement with refs (10, 32, and 35).

Figure 5 schematically illustrates the obtained results on relative ORR activities for the Pt alloys in acidic and alkaline media as a function of lattice parameters. It is noted that the pseudo volcano plot depicted with dashed lines only supports the conceptional relationship between the ORR performance and the Pt lattice constant relating strain effect and the influence of alkali cations at the electrode/electrolyte interface. The larger lattice parameter of bulk electrodes represents higher compressive strains on the surfaces of Pt or Pt-overlayers. Pt(pc) has slightly too strong binding energy between the Pt surface and the reaction intermediates. The existence of the alkali cations at the interface influences the ORR activities, which increase with the lower cation hydration energy. However, in the case of Pt_5_Gd and Pt_5_Y electrodes, which have larger lattice parameters and slightly weaker binding energies, the presence of alkali cations with higher solvation energy optimizes the binding energies on the compressed surfaces. Consequently, the ORR activity increases with increasing hydration energy for Pt_5_Gd and Pt_5_Y electrodes. In the future outlook, the different ORR trends between the Pt-alloys and Pt(pc) electrodes due to the existence of different cations at interfaces can be further investigated by combining in situ EIS and Raman spectroscopy to identify the double-layer capacitance, the adsorption processes, and the surface chemical speciation, and to provide more comprehensive insights into the rational design of electrocatalysts.

## Conclusions

In summary, this work explored the effect of electrolyte cation on the electrochemical ORR under different surface strains of Pt-based electrocatalysts in alkaline solutions. It is revealed that the ORR activities of the studied electrodes follow the order of P_5_Gd > Pt_5_Y > Pt(pc) in acidic media. Compressive strains allow one to achieve more optimal binding energies for reaction intermediates than for the pure Pt surface, leading to enhanced ORR kinetics. Moreover, the ORR performance of the Pt alloys improves with the increase of cation hydration energy in the order of Li^+^> Na^+^ > K^+^ > Rb^+^ > Cs^+^; however, the pure Pt electrodes demonstrate the opposite trend in alkaline media. Our computational results support the experimental findings that the noncovalent interactions between the solvated cations and intermediates and the strain effects on the surfaces strongly influence the ORR overpotential. For a pure Pt surface, slightly too strong adsorption of reaction intermediates and the presence of structure-making alkali cations result in increased ORR overpotentials. In comparison, the presence of cations with a higher hydration energy optimizes the binding conditions of adsorbed species on the compressively strained surface, thus enhancing the ORR activities.

## Supporting Information Available

The X-ray diffraction (XRD) patterns of Pt_5_Gd, Pt_5_Y, and Pt polycrystalline electrodes; the XRD fitting results with the corresponding literature; X-ray photoelectron spectroscopy data; the typical cyclic voltammograms; the *iR*-corrected voltammograms of the electrodes in O_2_-saturated electrolytes; HER calibration data; calculated adsorption energies and corresponding oxygen reduction reaction overpotentials on the pristine Pt(111); calculated adsorption energies and corresponding ORR overpotentials on Pt(111) in the presence of alkali metal cations (PDF)
