# Supplementary material for: Influence of Alkali Metal Cations on the Oxygen Reduction Activity of Pt5Y and Pt5Gd Alloys
Source: J Phys Chem C Nanomater Interfaces. 2024 Mar 18;128(12):4969–77. doi: 10.1021/acs.jpcc.4c00531 (PMC10983829; doi:10.1021/acs.jpcc.4c00531)
Supplement: Supplementary file 1 — jp4c00531_si_001.pdf [file jp4c00531_si_001.pdf]

Supporting Information:

Influence of Alkali Metal Cations on the Oxygen Reduction Activity of Pt_5Y and Pt_5Gd Alloys

Kun-Ting Song, [a] *Alexandra Zagalskaya,*[b,c] *Christian M. Schott,* [a] *Peter M. Schneider,* [a] *Batyr Garlyyev,* [a] *Vitaly Alexandrov,* [b,d] *Aliaksandr S. Bandarenka* [a,e,]*

a - Physik-Department ECS, Technische Universität München, James-Franck-Str. 1, D-85748 Garching, Germany.

b - Department of Chemical and Biomolecular Engineering, University of Nebraska-Lincoln, Lincoln, Nebraska 68588, United States

c - Quantum Simulations Group, Materials Science Division, Lawrence Livermore National Laboratory, Livermore, CA 94550, United States

d - Nebraska Center for Materials and Nanoscience, University of Nebraska-Lincoln, Lincoln, Nebraska 68588, United States

e - Catalysis Research Center TUM, Ernst-Otto-Fischer-Straße 1, 85748 Garching bei München, Germany

Keywords:

Platinum-based catalysts, oxygen reduction reaction (ORR), alkali metal cations, strain

Corresponding Author: Tel. +49 (0) 89 289 12531, E-mail: bandarenka@ph.tum.de (A.S. Bandarenka)

(A) (B) (C)

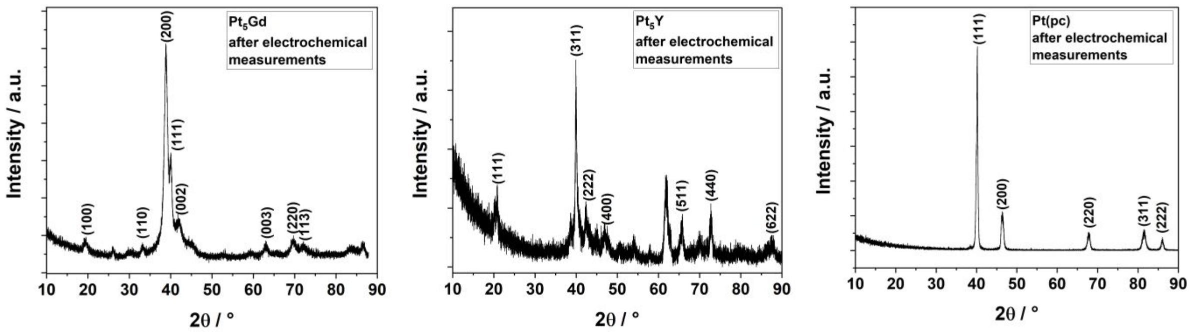

Figure S1. *The X-ray diffraction (XRD) patterns of (A) Pt_5Gd, (B) Pt_5Y, and (C) Pt polycrystalline (Pt(pc)) electrodes with labeled peaks correspond to the literature [1,2,3]. It is noted that all spectrums were recorded after electrochemical measurements.*

Table S1. *The XRD fitting results with the corresponding literature.*

Crystal electrode	Unit cell	Crystallographic prototypes	Lattice parameter a-axis [Å]	Corresponding literature
Pt_5Gd	FCC	Cu_5Ca	~5.29	[1],[3]
Pt_5Y	FCC	$AuBe_5$	~7.49	[2]
Pt	FCC	Cu	~3.92	[2],[3]

(A)

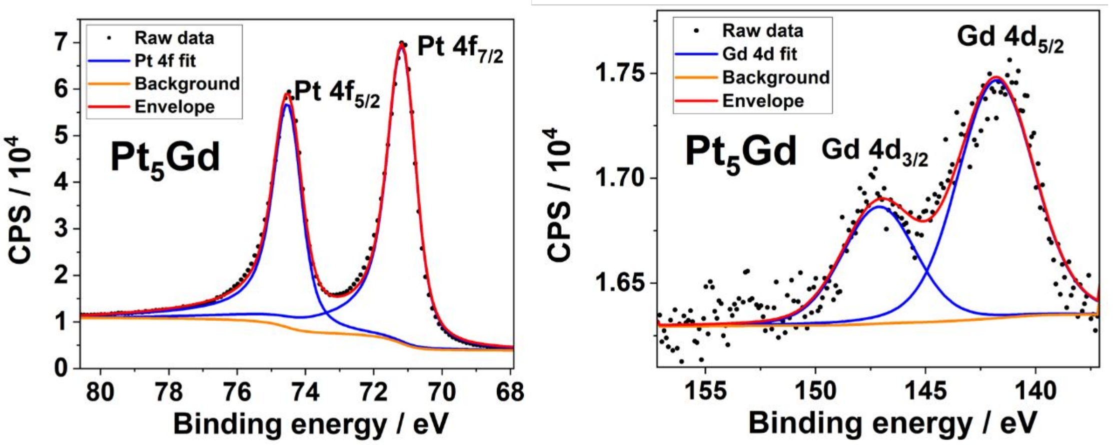

(B)

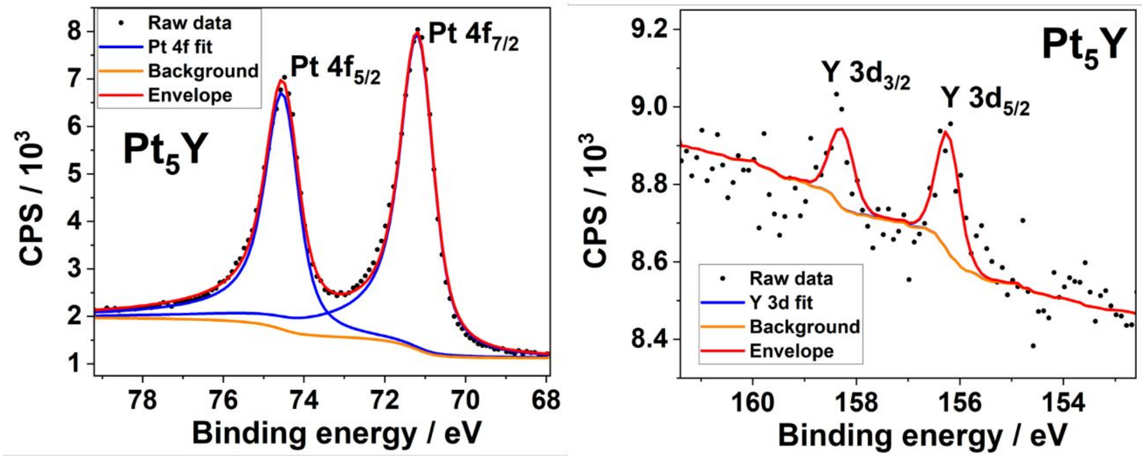

Figure S2. X-ray photoelectron spectroscopy (*XPS) data of (A) Pt_5Gd and (B) Pt_5Y electrodes after the electrochemical measurements. The fitting XPS results show a ~6.2 % Gd and ~4.2% Y for Pt_5Gd and Pt_5Y electrodes, respectively, which indicates a completed dealloying process on the surfaces.*

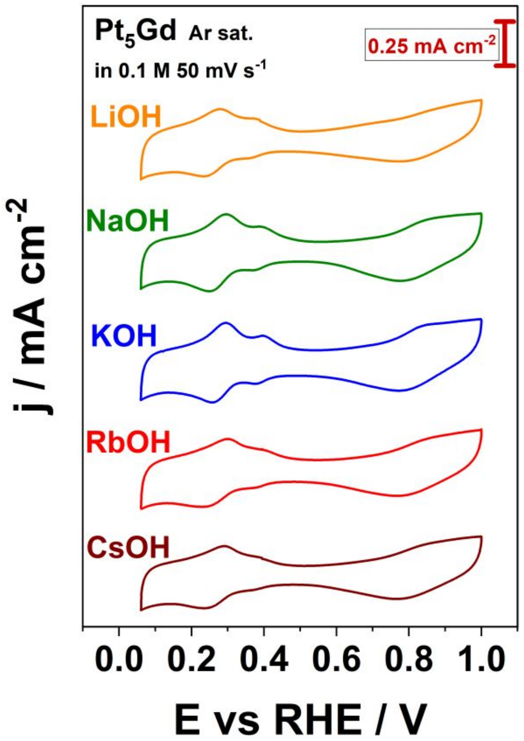

Figure S3. *The typical cyclic voltammograms for Pt_5Gd electrodes in Ar-saturated 0.1 M AM−OH (AM = Li^+, Na^+, K^+, Rb^+, and Cs^+) electrolytes with a scan rate of 50 mV s^{-1}.*

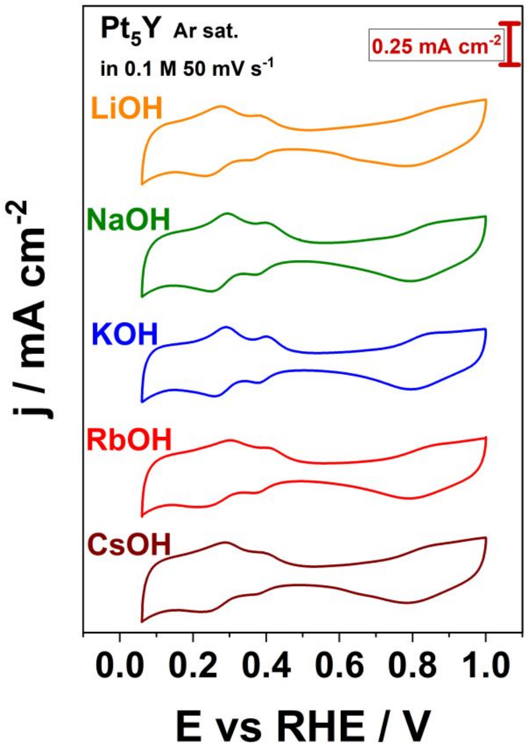

Figure S4. *The typical cyclic voltammograms for Pt_5Y electrodes in Ar-saturated 0.1 M AM−OH (AM = Li^+, Na^+, K^+, Rb^+, and Cs^+) electrolytes with a scan rate of 50 mV s^{-1}.*

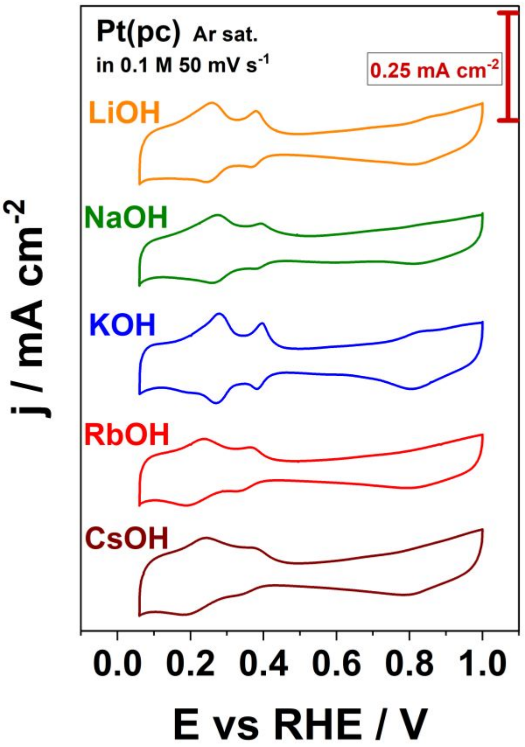

Figure S5. *The typical cyclic voltammograms for Pt(pc) electrodes in Ar-saturated 0.1 M AM−OH (AM = Li^+, Na^+, K^+, Rb^+, and Cs^+) electrolytes with a scan rate of 50 mV s^{-1}.*

(A)

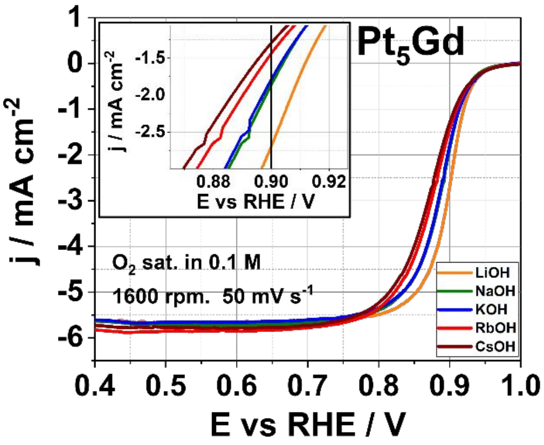

(B)

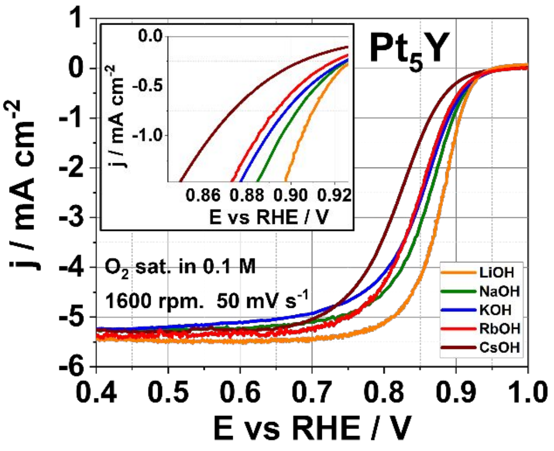

(C)

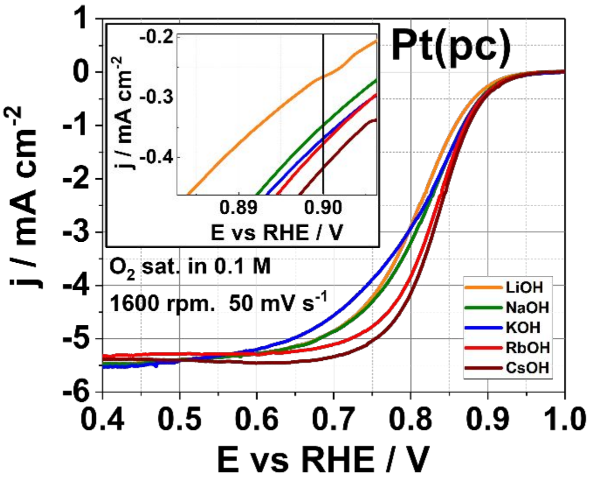

Figure S6. *The iR-corrected voltammograms with the anodic scans of (A) Pt_5Gd, (B) Pt_5Y, and (C) Pt(pc) electrodes in O_2-saturated 0.1 M AM−OH (AM = Li^+, Na^+, K^+, Rb^+, and Cs^+) electrolytes with the scan rate of 50 mV s^{-1} at 1600 rpm. The insets represent the* "zoomed-in" view of *voltammograms within the potential at about 0.9 V vs the reversible hydrogen electrode (RHE) scale.*

(A) (B)

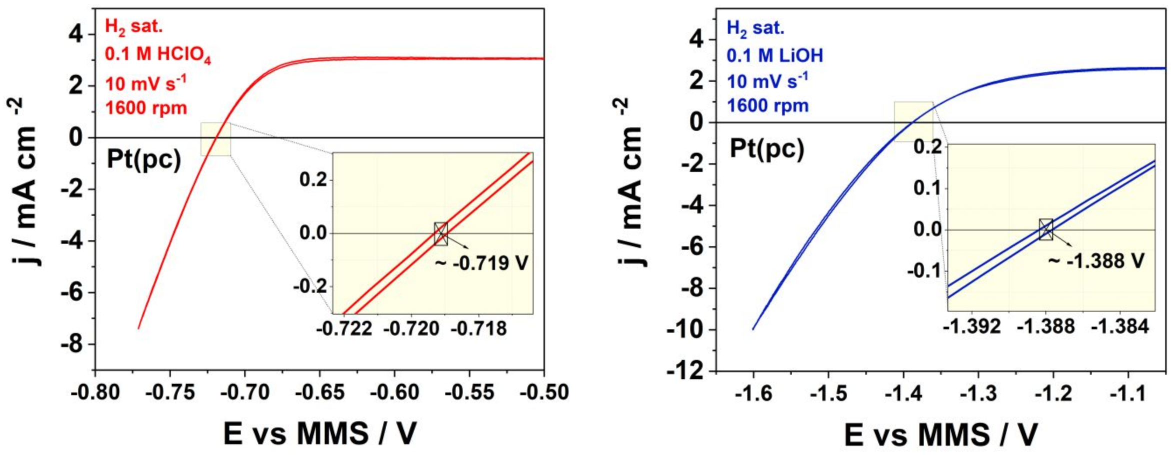

Figure S7. *HER calibration for Pt(pc) electrode in H_2-saturated 0.1 M (A) $HClO_4$ and (B) LiOH at the scan rate of 10 mV s^{-1} at 1600 rpm with the insets of potential close to interception region.*

(A) (B)

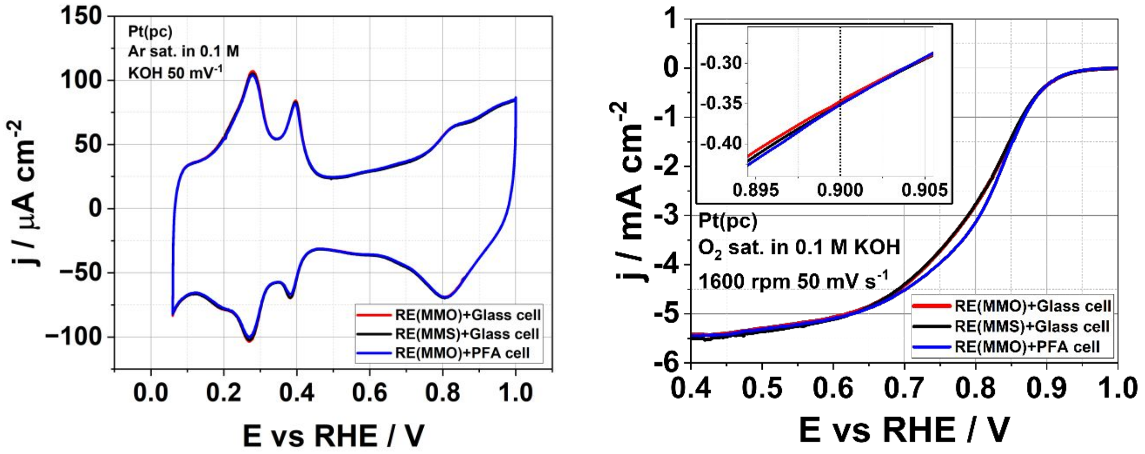

Figure S8*. (A)The cyclic voltammograms for Pt(pc) in Ar saturated 0.1 M KOH and (B) the iR-corrected oxygen reduction reaction (ORR) voltammograms with the anodic scan for Pt(pc) in O_2-saturated 0.1 M KOH with a scan rate of 50 mV s^{-1} by using mercury-mercurous sulfate (MMS) and mercury-mercuric oxide (MMO) as the reference electrodes and with the glass cell and the perfluoroalkoxy (PFA) cell. The inset figure (B) presents the "zoom-in" voltammograms at a potential close to 0.9V vs RHE.*

To prevent contamination problems [4,5], the cells were cleaned with the so-called "Piranha solution" and the electrochemical measurements were conducted over a short-term period. **Figure S8** shows the negligible differences for the cyclic voltammograms (CVs) and the oxygen reduction reaction (ORR) activities at 0.9 V vs RHE, recorded in Ar-saturated and O_2-saturated conditions, within the cases of the glass cell and the perfluoroalkoxy (PFA) cell with mercury-mercurous sulfate (MMS) and mercury-mercuric oxide (MMO) as the reference electrodes, respectively. The CVs in alkaline media are comparable to the reference [6] in which the Polytetrafluoroethylene (PTFE) cell was used in the electrochemical measurements.

Table S2. *HER calibration with different solutions and reference electrodes in the electrochemical glass cells.*

Solution (0.1 M)	Average interception point (V)	Reference electrode
$HClO_4$	~ -0.719	MMS
LiOH	~ -1.388	MMS
NaOH	~ -1.392	MMS
KOH	~ -1.393	MMS
RbOH	~ -1.395	MMS
CsOH	~-1.395	MMS
KOH	~ -0.906	MMO

Table S3. *The Pt-Pt distance in the slabs corresponding to the applied strain.*

Strain	-5.00%	-4.00%	-3.00%	-2.00%	0%	2%	4.00%
Pt-Pt distance, Å	2.687	2.715	2.743	2.772	2.828	2.885	2.941

Table S4. *Calculated adsorption energies and corresponding oxygen reduction reaction (ORR) overpotentials on the pristine Pt(111).*

Strain	ΔG(OH)-ΔG(OH)Pt@0%	ΔGads(O), eV	ΔGads(OH), eV	ΔGads(OOH), eV	ΔG1, eV	ΔG2, eV	ΔG3, eV	ΔG4, eV	**η, V**
Pristine Pt(111)									
-5%	0.19	3.04	1.54	4.56	-0.36	-1.52	-1.50	-1.54	**0.87**
-4%	0.15	2.98	1.50	4.52	-0.40	-1.54	-1.48	-1.50	**0.83**
-3%	0.11	2.92	1.46	4.47	-0.45	-1.55	-1.46	-1.46	**0.78**
-2%	0.07	2.12	1.42	4.43	-0.49	-2.31	-0.70	-1.42	**0.74**
0%	0.00	2.75	1.35	4.35	-0.57	-1.59	-1.40	-1.35	**0.66**
2%	-0.05	2.65	1.30	4.30	-0.62	-1.65	-1.35	-1.30	**0.61**
4%	-0.13	2.55	1.22	4.21	-0.71	-1.65	-1.33	-1.22	**0.52**

Table S5. *Calculated adsorption energies and corresponding ORR overpotentials on Pt(111) in the presence of alkali metal cations.*

Strain	ΔG(OH)-ΔG(OH)Pt@0%	ΔGads(O), eV	ΔGads(OH), eV	ΔGads(OOH), eV	ΔG1, eV	ΔG2, eV	ΔG3, eV	ΔG4, eV	**η, V**
Li^+@Pt(111)									
-5%	0.15	2.18	0.75	3.86	-1.06	-1.68	-1.43	-0.75	**0.48**
-4%	0.12	2.14	0.72	3.83	-1.09	-1.68	-1.42	-0.72	**0.51**
-3%	0.09	2.11	0.69	3.80	-1.12	-1.70	-1.42	-0.69	**0.54**
-2%	0.06	1.67	0.66	3.77	-1.15	-2.10	-1.01	-0.66	**0.57**
0%	0.00	1.99	0.60	3.70	-1.22	-1.71	-1.40	-0.60	**0.63**
2%	-0.05	1.90	0.54	3.65	-1.27	-1.76	-1.35	-0.54	**0.69**
4%	-0.09	1.86	0.51	3.61	-1.31	-1.75	-1.36	-0.51	**0.72**
Na^+@Pt(111)									
-5%	0.19	2.40	0.99	4.04	-0.88	-1.64	-1.42	-0.99	**0.35**
-4%	0.17	2.36	0.96	4.02	-0.90	-1.66	-1.40	-0.96	**0.33**
-3%	0.14	2.32	0.93	3.99	-0.93	-1.66	-1.39	-0.93	**0.30**
-2%	0.09	2.06	0.89	3.94	-0.98	-1.89	-1.17	-0.89	**0.34**
0%	0.00	2.18	0.79	3.86	-1.06	-1.68	-1.38	-0.79	**0.44**
2%	-0.03	2.09	0.77	3.82	-1.10	-1.72	-1.32	-0.77	**0.46**
4%	-0.06	2.07	0.73	3.78	-1.14	-1.70	-1.34	-0.73	**0.50**
K^+@Pt(111)									
-5%	0.16	2.46	1.08	4.10	-0.82	-1.64	-1.38	-1.08	**0.41**
-4%	0.13	2.42	1.05	4.07	-0.85	-1.65	-1.37	-1.05	**0.38**
-3%	0.10	2.38	1.02	4.04	-0.88	-1.66	-1.36	-1.02	**0.35**
-2%	0.07	2.03	0.98	4.01	-0.91	-1.98	-1.04	-0.98	**0.32**
0%	0.00	2.25	0.91	3.93	-0.99	-1.68	-1.34	-0.91	**0.32**
2%	-0.06	2.15	0.86	3.88	-1.04	-1.73	-1.29	-0.86	**0.37**
4%	-0.10	2.10	0.81	3.82	-1.10	-1.71	-1.29	-0.81	**0.42**
Rb^+@Pt(111)									
-5%	0.17	2.48	1.12	4.13	-0.79	-1.65	-1.37	-1.12	**0.44**
-4%	0.13	2.44	1.08	4.10	-0.82	-1.65	-1.36	-1.08	**0.41**
-3%	0.10	2.40	1.05	4.06	-0.86	-1.66	-1.36	-1.05	**0.37**
-2%	0.07	2.36	1.01	4.03	-0.89	-1.67	-1.35	-1.01	**0.34**
0%	0.00	2.28	0.95	3.96	-0.96	-1.68	-1.33	-0.95	**0.28**
2%	-0.06	2.17	0.89	3.90	-1.02	-1.73	-1.28	-0.89	**0.34**
4%	-0.10	2.12	0.85	3.84	-1.08	-1.72	-1.28	-0.85	**0.38**
Cs^+@Pt(111)									
-5%	0.17	2.49	1.15	4.16	-0.76	-1.67	-1.34	-1.15	**0.47**
-4%	0.13	2.45	1.11	4.13	-0.79	-1.68	-1.33	-1.11	**0.44**
-3%	0.10	2.41	1.08	4.10	-0.82	-1.68	-1.34	-1.08	**0.41**
-2%	0.07	2.03	1.05	4.06	-0.86	-2.03	-0.99	-1.05	**0.37**
0%	0.00	2.29	0.98	3.99	-0.93	-1.71	-1.31	-0.98	**0.30**
2%	-0.06	2.18	0.92	3.94	-0.98	-1.75	-1.26	-0.92	**0.31**
4%	-0.10	2.14	0.88	3.88	-1.04	-1.74	-1.26	-0.88	**0.35**

References

[1] Escudero-Escribano, M.; Malacrida, P.; Hansen, M. H.; Vej-Hansen, U. G.; Velázquez-Palenzuela, A.; Tripkovic, V.; Schiøtz, J.; Rossmeisl, J.; Stephens, I. E. L.; Chorkendorff, I. Tuning the Activity of Pt Alloy Electrocatalysts by Means of the Lanthanide Contraction. *Science* **2016**, *352* (6281), 73–76

[2] Stephens, I. E. L.; Bondarenko, A. S.; Bech, L.; Chorkendorff, I. Oxygen Electroreduction Activity and X-ray Photoelectron Spectroscopy of Platinum and Early Transition Metal Alloys. *ChemCatChem* **2012**, *4* (3), 341–349.

[3] Escudero-Escribano, M.; Verdaguer-Casadevall, A.; Malacrida, P.; Grønbjerg, U.; Knudsen, B. P.; Jepsen, A. K.; Rossmeisl, J.; Stephens, I. E. L.; Chorkendorff, I. Pt5Gd as a Highly Active and Stable Catalyst for Oxygen Electroreduction. *J. Am. Chem. Soc.* **2012**, *134* (40), 16476–16479.

[4] Mayrhofer, K. J. J.; Crampton, A. S.; Wiberg, G. K. H.; Arenz, M. Analysis of the Impact of Individual Glass Constituents on Electrocatalysis on Pt Electrodes in Alkaline Solution. *J. Electrochem. Soc.* **2008**, *155* (6), P78.

[5] Sebastián-Pascual, P.; Sarabia, F. J.; Climent, V.; Feliu, J. M.; Escudero-Escribano, M. Elucidating the Structure of the Cu-Alkaline Electrochemical Interface with the Laser-Induced Temperature Jump Method. *J. Phys. Chem. C* **2020**, *124* (42), 23253–23259.

[6] Rheinländer, P.; Henning, S.; Herranz, J.; Gasteiger, H. A. Comparing Hydrogen Oxidation and Evolution Reaction Kinetics on Polycrystalline Platinum in 0.1 M and 1 M KOH. *ECS Trans.* **2013**, *50* (2), 2163.
